# Transitional journeys into, and through medical education for First-in-Family (FiF) students: a qualitative interview study

**DOI:** 10.1186/s12909-018-1217-z

**Published:** 2018-05-09

**Authors:** Andrew Mark Bassett, Caragh Brosnan, Erica Southgate, Heidi Lempp

**Affiliations:** 1 Kings College, London, Weston Education Centre, Third Floor-Room 3.52, 10 Cutcombe Road, London, SE5 9RJ UK; 2 The University of Newcastle Australia, Room 349, Behavioural Sciences, Callaghan University Drive, Callaghan, NSW 2308 Australia; 3 The University of Newcastle Australia, Room HC52, Hunter Building, Callaghan University Drive, Callaghan, NSW 2308 Australia

**Keywords:** Qualitative, First-in-Family, Medical Students, Widening Participation, Transitions, Medical Education, Classism, Social Mobility

## Abstract

**Background:**

There has been much interest in the transitions along the medical education continuum. However, little is known about how students from non-traditional backgrounds experience both the move to, and through Medical School, and their ambitions post-graduation. This research sought to understand the transitional journey into, and through undergraduate medical education, and future career aspirations for first-in-family (FiF) medical students.

**Methods:**

Based on a interpretivist epistemological perspective, 20 FiF students from one English Medical School participated in semi-structured interviews. Participants were identified according to purposive inclusion criteria and were contacted by email via the student association at the Medical School and academic year leaders. The team approach to the thematic analysis enhanced the findings credibility. This research was part of an international collaboration.

**Results:**

In the first transition, ‘The Road to Medical School’, a passion for science with an interest in people was a motivator to study medicine. Participants’ parents’ shared the elation of acceptance into Medical School, however, the support from school/college teachers was a mixed experience. In ‘The Medical School Journey’ transition, knowledge about the medical curriculum was variable. ‘Fitting’ in at Medical School was a problem for some, but studying for an elite degree elevated social status for many study participants. A source of support derived from senior medical student peers, but a medical degree could sacrifice students’ own health. In the final transition, ‘Future Plans’, a medical career was perceived to have intrinsic value. Clarity about future aspirations was related to clinical experience. For some, career trajectories were related to a work-life balance and future NHS working conditions for Junior Doctors.

**Conclusions:**

The transitions highlighted in this article have important implications for those educators interested in a life cycle approach to widening participation in medical education. Future research should explore the post-graduation transitions for doctors from first-in-family University backgrounds.

## Background

### Access Barriers to Medical School: Experiences of Non-Traditional Students

Since the late 1970’s [1] the rationale for fairer access to undergraduate medical education has gathered internationally. Reflective of its rigid social class structure, the policy emphasis in the UK has been on growing the proportion of medical students from low socio-economic circumstances [2–7]. A retrospective analysis of applications to 22 UK Medical Schools between 2009 and 2012 showed that 80% of medical students were from professional households [8].

Qualitative research in the UK has illuminated the difficulties of applying to, and accessing undergraduate medical education for students from non-traditional University backgrounds. For instance, research pointed out the poor support of schools, and the active discouragement of teachers to study medicine [9–11]. Canadian studies have shown that financial worries and the cost of a medical education were access barriers to Medical School for non-traditional students [12, 13]. An Australian study of academically able high school pupils from low socio-economic circumstances, identified a lack of opportunities to undertake work experience in the health professions [14].

### At Medical School: Working Class, Low Socio-Economic, First-in-Family Students’ Perspectives

A limited amount of research exists on the experiences of undergraduate medical education for students from non-traditional University backgrounds. Qualitative research at a Canadian Medical School highlighted the common experience of ‘classism’ for working class medical students [15, 16]. Classism involves the perpetuation of hierarchies of social class via “everyday, common place practices” [16]. This manifested itself in the ‘othering’ and stigmatisation of working class students by peers and clinical staff [15, 16].

A more recent qualitative study [17] in a UK Medical School suggested that students from low socio-economic backgrounds struggled to engage with the ‘medical habitus’ and form a professional identity. An Australian study [18] used Bourdieu’s forms of capital to understand the perspectives of first-in-family (FiF) medical students. These students lacked the social networks to help progress through, and pursue future career aspirations at Medical School.

### Medical Education Transitions: First-in-Family (FiF) Medical Students’ Experiences

The extant research has considered the barriers to [9–14, 19, 20], or experiences at Medical School for students with non-traditional backgrounds [15–18, 21, 22]. However, to our knowledge, no UK studies have examined the transitional experiences both into, and through undergraduate medical education, and future career aspirations for first-in-family (FiF) medical students. This is despite sustained interest in the transitions that occur across the medical continuum for medical students as a whole [23, 24]. A transition is not a static point in time, but an open process whereby an individual undergoes a shift “from one set of circumstances to another” [23]. For medical educators, transitions are of significance, as individuals experiencing change will invariably encounter “new challenges, opportunities, stress and a range of emotions” [23]. Important, is how individuals adapt to, and cope with the demands of transitions [23]. To broaden the scope of equality of opportunity in medical education from a narrow widening access approach to a life cycle one [25–27], the experiences generated by transitions for first-in-family (FiF) medical students requires further understanding.

Our study is the second piece of research from an international collaboration on the transitions into, and through undergraduate medical education, as well as the aspirations for first-in-family (FiF) medical students. First-in-family (FiF) is defined as a medical student without a parent who studied in higher education [18, 28, 29]. The category of FiF not only conveys the experiences of those from low socio-economic backgrounds, but also for students where higher education is an unfamiliar terrain due to familial circumstances [18].

The first study by Southgate et al. [29] was a qualitative interview study of 22 FiF medical students at an Australian Medical School. Personal doubts about “not being good enough” [29] to train as a medical doctor was a psychological barrier to accessing Medical School. At Medical School, the FiF students perceived a social, economic, and symbolic separation between themselves and privileged classmates. This included differences in accent, financial circumstances, educational background before Medical School, and rural vs. urban upbringing. This study also explored what it meant to practice medicine post-graduation. Whilst the financial rewards of a doctor were recognised, the moral responsibility attached to the profession was deemed important.

Southgate’s et al. [29] study provided an invaluable insight into FiF medical students’ transitional and upwardly social mobile journeys into and through undergraduate medical education and aspirations post-graduation. However, this study was conducted in Australia and reflects the social inequalities of its context, including the transitional difficulties faced by Indigenous students. Using the concept of transitions [23], our study attempted to understand three important changes undertaken by FiF students at one English Medical School in their medical education journeys. The research objectives included:

1. to explore the transition into undergraduate medical education for FiF medical students (the pre-entry transition);

2. to understand FiF students’ experiences of undergraduate medical education (the period in Medical School transition);

3. to examine the aspirations of FiF medical students after degree completion (the prospective post-graduation transition).

The results of this study from an English Medical School can be pooled together with the previous FiF medical student study in Australia [29] and future findings of our international collaborators to create an international perspective on FiF students’ transitions into, and through medical education.

## Methods

### Methodology

As part of an international study of FiF medical students’ experiences, our research replicated as closely as possible Southgate et al.’s [29] methodological approach. A qualitative study design [30] fitted in well with the focus on the transitional experiences of FiF medical students. Congruent with this approach was an interpretivist epistemology and constructionist ontology [31]. An interpretivist perspective seeks to “gain meaning and understanding from situations and actions by interpretations and explanations of behaviours”, instead of the positivist establishment of “cause and effect relationships” [32]. A constructionist ontology emphasises how meanings and social phenomena are in a perpetual state of flux as they are acted out in social interaction [32]. Ethical approval was granted (30.11.2016) by one UK Higher Education Institution (name can be supplied by first author) .

### Sample Design/Recruitment

Participants were recruited from one English Medical School, which has both a five year Bachelor of Medicine, Bachelor of Surgery (MBBS), and a six year extended medical degree. The extended course has up to 50 students per academic year, uses contextualised selection criteria, and only admits applicants from non-selective state schools [33]. The first two academic years for the MBBS are pre-clinical (Years 1 & 2), whereas for the extended degree, it is the first three years (Years 1, 2 & 3). After completion of the pre-clinical stage, students in both medical degree programmes (MBBS Years 3–5; Extended Degree Years 4–6) proceed onto three clinical years of study. Latest institutional data [33] for 2015/16, indicated that 354 Home and European Union (EU) students were admitted to both five and six year degrees.

Given the interpretive orientation of the study, purposive criterion sampling was used to select research participants [34]. This strategy identifies participants according to specified inclusion criteria based on the study purposes and aims [35]. The research applied two inclusion criteria: (i) self-identified FiF status (ii); and from all academic years of the five and six year medical degree programmes. The ‘Medical Student Association’ at the University and the MBBS and extended degree academic year leaders emailed all their students an information letter, inviting them to participate if they identified as students with a first-in-family (FiF) University background (see definition above). The study budget permitted the recruitment of 20 FiF medical students. This was similar to the number of FiF participants in Southgate et al.’s study [29], and was sufficient to identify relevant themes and subthemes [36] in the dataset.

### Interview Schedule/Data Collection

Semi-structured interviews [37] were conducted with 20 FiF medical students that allowed the study authors to focus the questions around the three transitions, based on the research objectives. The interview schedule drew on the earlier Australian FiF medical student study [29], which strengthened its robustness. It included four topics: (i) the decision to study medicine and the Medical School application process; (ii) expectations and experiences of University and Medical School; (iii) barriers and facilitators to academic success; (iv) future goals and aspirations post-medical degree.

From December 2016 to March 2017, the first author arranged and conducted the interviews. At the start of each interview, a short socio-demographic questionnaire was completed by the participant. This contained screening questions on FiF status, age and gender, parental education and employment, Medical School entry route, type of medical degree (MBBS or extended degree), and year of academic study. To determine the relevance and appropriateness of the questions and study design [38] three pilot interviews were carried out. No modifications to the interview process and interview guide content were necessary, and therefore the three pilots were included in the main study.

The majority of interviews (*n* = 16) were face-to-face, but as some participants were on clinical placements, a few (*n* = 4) were conducted by telephone. Each participant received a £20 Amazon voucher as an appreciation of their time to participate. The average median duration of the interviews was 60 min. Written and informed consent was obtained prior to the audio recorded interviews which were then transcribed verbatim. The first author (AB) was from a social science and first-in-family University background, but had no prior academic or teaching experience in medical education. Thus, through personal experience, the first author had a close identification with many of the accounts shared by the study participants and how FiF status shaped the experience of higher education. This shared FiF background between interviewer and interviewee generated close rapport and helped in the generation of rich data. Through close supervision by the last author during the data analysis potential bias due to the shared background was mitigated.

### Analysis

Thematic analysis [39] was applied to the transcribed interviews with the computer software programme NVivo 11 (Melbourne, Australia). At the initial stage, the first three interview transcripts (participant 1–3) were independently and inductively coded [40] by the four study authors. From this inter-coding process a provisional framework emerged with three broad themes. In the second stage of analysis, the first and fourth author separately applied the framework to the fourth transcript (participant 4). Through a process of consensus the analysis was refined further. This collaborative approach ensured the consistency of themes and subthemes and achieved inter-coding reliability [41]. In the third stage, the first author used the framework to analyse the other 16 interviews by a constant comparative technique [39], which also meant being open to new inductive codes in the data. Finally, the fourth author provided feedback on the credibility of the first author’s interpretation of the data after the completion of analysis [42]. As AI-Busaidi [43] noted, having multiple analysts is a form of triangulation and is a useful technique of verification. The widening participation and wider medical education literature was employed to interpret the findings and was aided by the fact that the second, third, and fourth authors had academic backgrounds in medical education and a firm knowledge of the study area.

## Results

### Participants’ Characteristics

The two tables highlight the FiF participants’ socio-demographic characteristics (Tables 1 and 2).

**Table 1:** Social Characteristics of Participants

Gender No. (%)	Ethnic Background No. (%)	Age Group No. (%)	Participants’ Parental Employment Status No. (%)
-Male 8 (40) -Female 12 (60)	-White British 5 (20) -Asian British 5 (20) -Other Ethnic Background 5 (20) -Black African 2 (10) -Mixed Ethnicity 1 (5) -Caribbean 1 (5) -Asian 1(5)	−18-21 17 (85) − 22+ 3 (15)	-Housewife 6 (19.3) -Administration 6 (19.3) -Taxi driver 4 (12.9) -Retail 4 (12.9) -Small business owner 4 (12.9) -Care worker 3 (9.6) -Managerial 2 (6.4) -Retired 2 (6.4)
Total 20(100)	Total 20 (100)	Total 20 (100)	Total 31 (100)

**Table 2:** Educational Profile of Participants

Highest stage of education achieved by participants’ parent(s) No. (%)	Entry route into Undergraduate Medical Education No. (%)	Medical Degree Type No. (%)	Medical Degree Stage No. (%)
- Primary Level 1 (5) - Secondary Level 14 (70) - Tertiary Level 5(25)	-Direct entry from Tertiary Education 15 (75) -Gap year between Tertiary Education and Medical School 2 (10) -Graduate Entry 3 (15)	-Extended (6 Years) 11 (55) -MBBS (5 Years) 9 (45)	-Pre-Clinical Stage 10 (50%) -Clinical Stage 10 (50%)
Total 20(100)	Total 20(100)	Total 20(100)	Total 20(100)

More females were represented in the sample than males. Available Medical School data [33] between 2003/04–2015/16, showed that the average representation for women on the traditional degree was 58.1% compared to 57.3% on the extended programme. One FiF study participant was an international student, whilst the rest of the interviewees were from the UK. The majority of participants derived from non-White British backgrounds (*n* = 15, 75%). Medical School data [33] over the period 2003/04–2015/16, indicated that 50% of the five year programme and 12.9% of the six year course had a White British or Other White ethnicity. Most participants enrolled at Medical School between the ages of 18–21. On average, from 2003/04–2015/16, 82.4% of students on entry to both medical degrees had the age range of 18–21 [33].

None of the FiF students came from medical family backgrounds. Rather, a large number of FiF students’ parents worked in routine/semi-routine occupations [44]. This included health and social care, retail, and taxi driving. There was no institutional data for students’ parents educational level or entry route into Medical School. However, our small sample of FiF students revealed that the majority of participants’ parents were educated to Secondary Level. In addition, the majority enrolled at Medical School after GCE Advanced Levels (A Levels). More participants were from the extended degree. Across the two degree types, equal proportions of FiF students were recruited from pre-clinical and clinical stages.

### Themes/Transitions from the Interviews

Several subthemes were identified within each of the transitions/themes. These transitions with their subthemes are shown in Table 3.

**Table 3:** Transitions and Subthemes

Transition 1: The Road to Medical School	Transition 2: The Medical School Journey	Transition 3 Future Plans
Subthemes • Motivations to Study Medicine • Familial and School/College Expectations to Study Medicine • Participants and Family Reactions to Acceptance into Medical School	Subthemes • Expectations of Medical School • ‘Fitting’ into the Socially Mobile World of Medicine • Personal Sacrifices of Studying Medicine • Informal/Formal Supports at Medical School	Subthemes • What it Means to Practice Medicine • Career Aspirations

The number (*n*=) of students who reported a particular subtheme of a transition/theme is highlighted in the text. Although it cannot provide the basis for statistical generalisation to the wider FiF student population in the Medical School, citing the frequency (*n*=) gives an indication of the prevalence of subthemes in the data set [45]. Individual accounts from interviews are used to illustrate the subthemes. Participants are identified by interview number, gender, year of academic study, and the type of medical degree studied (e.g. traditional or extended degree).

### Transition 1: ‘The Road to Medical School’

#### Motivations to Study Medicine

An important motivation to study medicine was the illness of a family member (*n* = 7). This compelled the students to apply the knowledge from a medical degree to help others in similar circumstances in the future:

"... after my GCSE's, my nan [grandmother] got really ill... I went into the hospital one day and I saw the kind of work that they [doctors] were doing... If it wasn't for them, my nan would not have been able to walk, because she has been a heart patient since she was 30... from then on, I was really sure that I wanted to do medicine and give something back to the NHS and to other people". (Participant 15, Female, Year 1 of Extended Degree)

Another reason was the perceived financial and job security (*n* = 5) of a career in medicine. However, the most cited reason (*n* = 17) to study medicine was a curiosity about science alongside an interest in working with people:

"... I like the idea of taking in, or learning a lot of science... And also having some sort of exposure to the community, with being able to talk to people and not being confined to a lab or some kind of research facility. I think that was a big plus for me to study medicine". (Participant 11, Male, Year 2 of Traditional Degree)

#### Familial and School/College Expectations to Study Medicine

The participants were asked about their family’s reaction to the decision to apply for Medical School. Whilst a majority (*n* = 18) of participants’ spoke of the moral support of relatives, six described a familial expectation linked to cultural background to study medicine:

"... being from a family from an East African background... in my country Somalia, there is a lack of infrastructure, solid government, health care system... my parents wanted me to do medicine, because that's what they lacked in their home country... if I was born into an English family, where all of this is available in the NHS, then perhaps, my parents would have given me more freedom to do something else..." (Participant 13, Male, Year 4 of Extended Degree)

A few participants (*n* = 7) recalled the active discouragement of some (but not all) school/college teachers to apply for Medical School. These FiF students were informed that they did not have the required intellectual prerequisites, and/or to consider less rigorous academic degrees, and/or that Medical School was too competitive to access:

"... they [teachers] say, 'you can't do that [study medicine], because it is too high for you'. They always give the story of the college, for that a lot of people have applied [to Medical School], but always fail, and that there was one person in the college history that got into medicine... One of my friends, who got three A-stars and one A [A Levels] wanted to go into medicine, but the college didn't give him any support. I remember the teachers laughing at him, because my friend had all these excellent predicted grades, but they said he was not getting in, because he didn't have the interview skills... But why didn't they help him gain those skills"? (Participant 17, Female, Year 2 of Traditional Degree)

Six of these seven participants referred to their own determination to fulfil their personal aspiration to get into Medical School and to prove the teachers’ doubts unjustified:

"... I needed to prove this particular teacher wrong, who was negative about [me] applying to medicine. So, I just relied on my own research on how to get into Medical School... looking at prospectuses, and just looking at endless information. I actually went back [after getting into Medical School] to this teacher and he was shocked. He could not even look me in the eye... that made me feel really happy inside as well". (Participant 3, Female, Year 3 of Extended Degree)

However, the majority (*n* = 17) of the FiF students’ mentioned that at least some teachers were supportive of their Medical School application. These teachers provided individual help including: organising medical related work experience; proof reading personal statements; interview preparation; guidance with the ‘United Kingdom Clinical Aptitude Test’ (UKCAT) and/or the ‘BioMedical Admissions Test’ (BMAT); and arranging visits to Medical Schools. Another type of support involved former pupils of the school/college, who were now medical students to give advice about Medical School:

"I remember that they [sixth-form] brought in one of their ex-students, who was doing medicine at Cambridge. And she [medical student] came in and talked to us about her experiences of Medical School". (Participant 8, Female, Year 6 of Extended Degree)

#### Participants and Family Reactions to Acceptance into Medical School

All participants’ (*n* = 20) recalled their reactions to the Medical School acceptance letter. This included: elation and/or relief; a sense of reward for hard work; and a fulfilment of a longstanding ambition. In the next account, a student described that they had defied the odds by entering Medical School:

"Well, I am working class. From my [working class] background, it is hard to make it into professions like medicine... I would say it has been an achievement for someone from my background to get to this stage [study medicine]". (Participant 16, Male, Year 1 of Extended Degree)

The FiF students (*n* = 20) gave accounts of the family’s response to the news of their acceptance into Medical School. Just one participant had an indifferent reaction from a parent. The others’ (*n* = 19) spoke of how (grand)parents shared the elation, were proud that their (grand)son or (grand)daughter was the first family member to get into higher education, and/or study an elite degree:

"... nobody has ever got into University in my family before... literally nobody... it was a massive thing for them. Like, I got into Medical School as well... they [family] were ecstatic". (Participant 9, Male, Year 1 of Traditional Degree)

### Transition 2: ‘The Medical School Journey’

#### Expectations of Medical School

Whilst some participants (*n* = 6) were aware of the general approach to learning at Medical School, others (*n* = 7) had no knowledge of the pre-clinical - clinical divide of the medical degree, or specific parts of the curriculum, including dissections, clinical skills, and histology and biochemistry:

"I didn't realise about the dissections... it is just the small details I did not realise we would do. Such as histology. I did not know that we did that stuff as part of the curriculum". (Participant 5, Female, Year 3 of Extended Degree)

The majority (*n* = 19) of participants had expected to experience problems adjusting to the academic demands of a medical degree. However, most (*n* = 15) had adapted to the Medical School’s reality of hard work and commitment. In the next account, a participant described how they doubted their academic ability until the first examination result:

"At the beginning it was hard. I mean, there was a reason they picked me to come here [Medical School], over other people that they rejected. So, I have to fit in to an extent... after my first exam in January I got a decent mark. I got 64% in my first January exam. I thought, yeah, perhaps I'm cut out for this. So it was after that January that I started to feel better". (Participant 13, Male, Year 4 of Extended Degree)

The others (*n* = 5) continued to face difficulties with the medical degree and questioned their academic capabilities to complete the course:

"... in the first year of Medical School I struggled a lot [with the course]. Like, I passed my exams, but I was never passing them that well. And that scared me. I remember talking to my personal tutor about how I couldn't cope [with degree] and that I was worried about being kicked out [of Medical School]... I do feel that I have to work really hard in order to stay here. Not saying that anyone else does not work hard, it is just that I do feel it has been a struggle". (Participant 10, Female, Year 4 of Extended Degree)

#### ‘Fitting’ into the Socially Mobile World of Medicine

Over a quarter (*n* = 6) of the FiF students mentioned that they had felt out of place around medical students from middle class and traditional University backgrounds:

"Everyone [other medical students] was posh in how they spoke. Just little differences in the way that they spoke was different to me. My first University [mature student] wasn't prestigious. And all the graduates [medical students] here had gone to Kings College, Cambridge, and Oxford... Sometimes, I felt like I wasn't supposed to be here [Medical School]. That I had got in on a whim". (Participant 12, Female, Year 2 of Traditional Degree)

One suggested he only became aware of his working class identity since starting Medical School:

"... I am from a working class background. But it was not until I came here to Medical School did I think about my social class. Because, before I came here [Medical School] no one was any different to me... I had never met people [before Medical School] who had parents, who had degrees from Cambridge and had gone on holidays to the Bahamas. I thought that I am from a different social class from these people". (Participant 9, Male, Year 1 of Traditional Degree)

Although instances of overt ‘othering’ and alienation of FiF students’ social and educational backgrounds by other medical students were uncommon, several occurrences (*n* = 3) were described:

"... a lot of the medical students here come from private schools... other medical students are surprised when I tell them that I came from a non-selective comprehensive school. They were very surprised by that. There was even a bit of felt disdain towards that [state school background]... It is their problem that I did not go to a private school..." (Participant 18, Male, Year 4 of Traditional Degree)

Despite a medical student identity and its connotations of an elite degree status, four participants’ asserted that their social class remained rooted to the family’s working class background. However, eight of the FiF students’ believed that studying medicine had elevated their social status, and this became apparent from how people outside of the institution reacted to them:

"People put it up [studying medicine] high, because, from where I grew up, no one really does medicine... [to] become a medical student is seen as quite prestigious, because we are going to become doctors". (Participant 12, Female, Year 2 of Traditional Degree)

#### Personal Sacrifices of Studying Medicine

Nearly all participants (*n* = 19) spoke of the constraints that the work load of a medical degree placed on their personal time. This sometimes created tensions with family and/or friends:

" ... if I do get some free time [from academic studies], I close myself off from a lot of people... that is because I am so tired... when I do get together with my friends and they are talking about all the [good] times they had, I was never there... it has even [Medical School] affected my family... if they want to go on a family holiday, it has to be arranged around me..." (Participant 10, Female, Year 4 of Extended Degree)

As well as the impact on time spent away from studies, eight of the FiF students had sacrificed their own health and well-being at some point in the degree. The combination of Medical School and paid-work, and/or the competitive nature of medical student academic life contributed to stress. In the following account, a FiF student described his experiences of burn out and fatigue:

"I could spend more time doing other things [on personal time], but I prioritise my studies over everything. I don't know if that is very healthy sometimes? Social life can definitely be affected. I think your own health can be affected... there is a lot of stress. An unhealthy amount of stress". (Participant 11, Male, Year 2 of Traditional Degree)

#### Informal/Formal Supports at Medical School

Many participants (*n* = 13) accessed formal pastoral and academic support from tutors, clinical advisors, and University professional services. However, the most cited form of support was provided by medical student peers (*n* = 17). This involved mutual help with revision and Objective Structured Clinical Examination (OSCE) preparation, or asking a fellow student to clarify a particular topic. Several students (*n* = 3) suggested that they would rather seek the advice of peers than seniors in clinics, because of a fear that their clinical competency would be questioned and help-seeking penalised. One reported that this was a common perception among fellow peers:

"... if I confide in my clinical advisor, then I don't know where that information is going to go, or where it will end up. I don't want to be negatively penalised for it... a lot of other people on the course feel exactly the same with their clinical supervisors. You know? That I don't trust this person [clinical supervisor] well enough to trust them with any sort of sensitive information". (Participant 18, Male, Year 4 of Traditional Degree)

Six participants fostered social ties with students in later years of Medical School. This provided invaluable academic advice:

"The help from the older years [medical students] is very helpful, because they are straight up with you. Because a teacher is like, when it comes to revision; 'you should go through everything and cover everything'... Whereas, they [senior medical students] are like; 'you have got 100 lectures to cover before the mid-session exam in January... you have to be smart about this. What do you need to prioritise more? What are your strengths and weaknesses'... this is the older year students giving us this advice". (Participant 4, Female, Year 3 of Extended Degree)

Although many (*n* = 11) turned to family for moral support, their medical student friends were more likely to relate to emotionally challenging experiences encountered at Medical School:

"... my friends here at University are very important, especially when providing support when you are down and stuff... they are just so encouraging as you are all in the same boat... [if] I can't cope, or it [academic demands] is getting too much, they [medical student friends] will sit down and have a plan... they are just there for you all the time". (Participant 3, Female, Year 3 of Extended Degree)

### Transition 3: ‘Future plans’

#### What It Means to Practice Medicine

For many participants (*n* = 12), a future career in medicine was a vocation or ‘calling’, which would provide intrinsic reward and value. In this context, job satisfaction would result from the beneficial effects on the lives of patients, rather than just the financial opportunities of a medical career:

"... if you want to become a doctor, money is not why you want to become a doctor. Like, you can become a bank manager or an accountant and you can earn way more money. But the reason why you want to become a doctor is that you want to give back [to the National Health Service] and you want to help people and you want to save lives. It sounds clichéd, [but] being a doctor is basically about helping people". (Participant 3, Female, Year 3 of Extended Degree)

In addition, a ‘good’ doctor was defined as someone driven by continual professional development (*n* = 2), or as the following account shows, ‘person-centred’ (*n* = 3) in the approach to care:

"The difference between good doctors and not so good doctors is how you communicate with people. Giving people proper patient-centred care... listening to the patient, in terms of what they need and being empathetic..." (Participant 20, Male, Year 5 of Traditional Degree)

#### Career Aspirations

Being clear about future medical career aspirations was linked to the degree of clinical experience that a student had been exposed to (*n* = 18).Whilst pre-clinical participants did not have the experience of placement to make an informed judgement, those FiF students in the later clinical years, spoke about the role of clinical supervisors, senior doctors, and patients in career decision making:

"... when you are thinking about specialities, you have got a range of things that different people are suited to... it is also influenced by your personal experience as you go through clinics, in terms of who you meet and what you see... So, if you are having a horrible registrar when you on a placement, you are unlikely ever to do that [specialism]... that is something I found talking to doctors about their [undergraduate] training, why they are doing what they are doing. They say; 'I remember this patient', or 'I remember that doctor'. And that tends to push them one way or another [specialism]". (Participant 1, Female, Year 3 of Traditional Degree)

In February 2016, the UK Department of Health imposed a new contract on Junior Doctors. This has proved controversial for several reasons, including: uncertainties about the number of working hours per week and the subsequent impact on Junior Doctor salaries; and the potential negative effect on patient safety with the introduction of untested alterations to working hours [46]. Due to the new Junior Doctor Contract, a quarter (*n* = 5) of the FiF students expected to leave medicine on graduation or work as a medical practitioner abroad:

"... morale has been quite low among quite a few of us, in terms of the new Junior Doctor contract. In that case [with the new Junior Doctor Contract], I will finish the degree, but I may not work in medicine, rather than leave the degree early". (Participant 14, Female, Year 6 of Extended Degree)

"It depends on the political climate [decision to practice medicine in UK]. Canada is the same as Australia, in that is has slightly better working conditions for doctors..." (Participant 1, Female, Year 3 of Traditional Degree)

Nine participants linked their future medical career aspirations to a need for a work-life balance. Surgery and emergency medicine were seen to involve stress, long hours, and inordinate levels of clinical responsibility. In contrast, general practice seemed to fit more with a desire for a family life and would provide the opportunity to specialise and work part-time:

"... emergency medicine was another option in my mind. But in terms of the work-life balance and the commitment levels, I don't really fancy that... being a GP gives you that [work-life balance], and then you can become a GP with a special interest". (Participant 13, Male, Year 4 of Extended Degree)

## Discussion

### Comparisons with Previous Literature

For FiF students, an academic curiosity in science alongside an interest in people was the most cited reason to pursue medicine. Although instrumental reasons were stated by some FiF participants in our study, more of them were compelled to study medicine, because of a serious illness and/or death in the family. These students had the intention to apply the knowledge and expertise of a medical degree to help others in similar unfortunate circumstances.

None of the FiF students had parents in the medical profession who, could have influenced the decision to study medicine. Reay [47] has used the term ‘familial habitus’ to explain why young people tend to align future expectations with their family background. Yet, in virtually all cases, the study indicated that parents were supportive of their son or daughter’s aspiration to study medicine. Furthermore, many students with black and ethnic minority backgrounds reported a strong familial expectation to get into Medical School. Mathers and Parry’s [22] UK study of mature working class medical students also suggested that strong parental support arose from the denial of opportunity and the desire for their children not to experience the negative life events that they had faced in their lives.

Some FiF students’ initial doubts about their academic ability to succeed at Medical School were compounded by the disparaging comments of school and/or college teachers. Previous research showed that teachers can hold erroneous or stereotypical assumptions about the socio-economic background of medical students [11]. This may lead to the active discouragement of academically able students from non-traditional backgrounds to apply for medicine [10, 11, 29]. McHarg, Mattick and Knight [11] argued that a strong message needs to be conveyed to teachers in secondary and further education that, irrespective of socio-economic circumstances, academically able students should be encouraged to study medicine and to consider a potential career in medicine. To achieve this aim, UK Medical Schools need to target medical students and secondary and further education teachers to become ambassadors for undergraduate medical education [11].

The negativity of some teachers spurred on FiF participants’ to fulfil their aspiration to study medicine against the odds. Cleland and Medhi [10] noted that in negotiating the various barriers to accessing Medical School, resilience is an important characteristic of applicants from non-traditional backgrounds. They suggested [10] this priority will have implications for the (i) increased emphasis on resilience in practitioner’s well-being and support [48], and (ii) whether Medical School students should be recruited from those who demonstrate resilience. If this is the case, widening access may benefit the medical profession by “challenging the dominant discourse of meritocracy”, [10] which has dominated the widening access literature.

Our findings indicated that the elation of acceptance into Medical School was shared by FiF participants and their family members. Southgate et al. [29] found something similar in their study of FiF medical students at an Australian Medical School. However, in our UK study, there was a stronger familial sense of the accrual of social status with successful entry to an elite degree. Previous research has focused on the complexities of the admissions process for non-traditional students [19, 20]. However, despite their admissions success, some of the FiF participants did not have the requisite knowledge or ‘cultural capital’ [49] of what a medical degree involved, including the pre-clinical-clinical division and/or specific aspects of the curriculum. Contact with medical students prior to Medical School entry may provide the best means of accessing such information [25]. To have credibility, the source of information should have homophily with the recipient of the advice. In the context of widening participation, this would be a medical student from a FiF or other non-traditional University background.

Some FiF participants initially struggled to ‘fit in’ with other students at Medical School. There was a sense that a middle class identity was the norm. Studies have shown how students in higher education internalise their non-traditional University identity as a form of stigma [50, 51]. This translates into a feeling of being ashamed about social background [29] and attempts to either ‘hide’ it from classmates, or even to ‘pass’ off as middle class [50, 51]. However, as in Southgate et al.’s [29] Australian study of FiF students, overt instances of ‘classism’ [15, 16] were uncommon. Despite, this, those rare incidences of ‘othering’ and stigmatisation were distressing experiences for FiF students.

Tim Wilkinson [52] argued that more attention should focus on the hidden curriculum of students at Medical School. Although afforded less concern than the hidden curriculum constructed by staff and faculty, it is no less powerful in its alienating effects for students from non-traditional backgrounds. Although, like Southgate et al.’s Australian FiF study [29], we found that instances of ‘classism’ were rare, nevertheless it must be dealt with as part of the Medical School curriculum, similarly to how cultural competence is taught to medical students [18]. As with all aspects of the hidden curriculum, the recognition of ‘classism’ as a problem and therefore transparency is important [53].

Our research documented the restrictions on FiF students’ personal time, due to the intensity of academic life at Medical School. The tensions that this can create in personal relationships with family members and friends has also been reported in a UK qualitative study on the transition from medical student to pre-registration doctor [54]. Similarly, Beagan’s study [15, 16] at a Canadian Medical School reported a growing social isolation of working class students from their families, which grew wider as they progressed through the degree. In addition, the academic competition among medical students placed an extra stress burden on some FiF participants. Lempp and Seale [55] suggest that an important characteristic of the hidden curriculum at Medical School is the value accorded to competition rather than cooperation between students. The reluctance of some FiF participants to seek the help of clinical supervisors may reflect the hidden curriculum’s emphasis on the need to impress senior clinical staff to secure future prestigious positions [55].

‘Peer mentoring’ is a common widening participation approach in UK Medical Schools [26]. It is commonly offered in the first year of Medical School, whereby new students are mentored by their second year peers. The focus is often on orientation and socialisation, but this assistance can support new students on how to adapt to the learning requirements of higher education. However, this study showed that some FiF participants were informally and strategically networking with students in senior years to obtain academic advantages, including guidance on examination revision and preparation. Such informal help-seeking seemed to be an aspect of the hidden curriculum between students, or as Lempp and Seale [55] note, part of those “implicit rules to survive the institution”.

To the authors’ knowledge, no research exists on the future aspirations of non-traditional medical students. A significant issue for some interviewees was the effect of the new NHS Junior Doctor contract on career plans post-graduation. The finding that a quarter of FiF participants were considering leaving medicine on graduation, or giving serious thought to practicing abroad is reflected in a recent UK cross-sectional study [56] of the wider medical student population. Combined with the as yet unforeseen consequences of Brexit, the imposition of the new Junior Doctor contract could have detrimental effects on the future medical workforce in the National Health Service [57].

Work-life balance was another important consideration in FiF students’ prospective medical career choices. Two cross-sectional surveys of UK medical students, indicated that a satisfactory work-life balance was of great significance, and had more value than the perceived need for certain aptitude or skills, intellectual challenge, or the amount of patient contact time [58, 59]. In addition, this data suggested that the importance attached to work-life balance is eroding traditional gender differences in medical career trajectories. As in this study, these surveys [58, 59] reported that because of its perceived work-life balance, general practice is seen as a more attractive prospective career than previously desirable specialism’s such as surgery.

### Suggestions for Further Research

There are evident gaps in the literature on the post-graduation transitions of first-in-family (FiF) students. A substantial body of statistical data suggests that over the last sixty years, students from non-traditional University backgrounds continue to be underrepresented in admission rates to UK Medical Schools [2–8]. Recent UK evidence on social mobility into elite professions also indicated that the majority of the medical workforce come from families of a higher managerial or professional background [60, 61]. Therefore, we suggest that it is an imperative for research to examine the post-graduate transitions for those with a first-in-family or other non-traditional University background. This could be longitudinal in design with a focus on the transition of medical graduates to Junior Doctor or specialist trainee, and on the final journey from trainee to medical specialist. As well as presenting opportunities for personal development [23], those from FiF circumstances may experience new challenges and barriers in these post-graduate transitions. The identification of such difficulties could go some way towards a lifecycle approach [25–27], that considers widening participation from pre-entry to Medical School to all stages of the medical education continuum [24] .

### Strengths and Limitations of the Study

This study provided a first-hand and in-depth insight of the medical education transitions for FiF students at an English Medical School. Challenges, uncertainties, and opportunities presented themselves to the FiF students in all three stages [23] of their medical education journey. The study has some limitations. It was carried out at a single Medical School in England. However, the contextual depth of participants’ accounts should help medical educators to decide whether the findings are translatable to other Medical School settings. The study results can be combined with proceeding [29] and subsequent findings of our international collaborators. In addition, the research is cross-sectional in design and was largely based on participants’ retrospective accounts. Despite this weakness, the study is an important contribution to the limited research base on the medical education transitions for non-traditional students.

## Conclusions

To our knowledge, this is the first UK study to examine the pre-entry (to Medical School), undergraduate, and prospective transitions for first-in-family (FiF) medical students. Each of these transitions presented uncertainties and challenges, but also opportunities for the research participants. The issues highlighted in this study may contribute to the formation of a coordinated lifecycle approach [25–27] to widening participation in medical education. However, given the data [60, 61] on social mobility into the medical profession, future research may want to focus on the post-graduation transitions of first-in-family [FiF] medical doctors.

## Abbreviations

A: Levels GCE Advanced Levels
BMAT: BioMedical Admissions Test
FiF: First-in-Family
MBBS: Bachelor of Medicine, Bachelor of Surgery
NHS: National Health Service
OSCE: Objective Structured Clinical Examination
UK: United Kingdom
UKCAT: United Kingdom Clinical Aptitude Test
